# Affective disorders: eliminate WArning signs And REstore functioning: AWARE. Results from a randomized controlled multimodular intervention study targeting functioning in patients with affective disorders

**DOI:** 10.1017/S0033291724002526

**Published:** 2024-10

**Authors:** Rasmus Schwarz, Kamilla Woznica Miskowiak, Mie Skovmand Christensen, Lars Vedel Kessing, Maj Vinberg

**Affiliations:** 1 Mental Health Centre, The Early Multimodular Prevention and Intervention Research Institution (EMPIRI), Hillerød, Denmark; 2 Copenhagen University Hospital – Mental Health Services CPH, Copenhagen, Denmark; 3 Department of Clinical Medicine, University of Copenhagen, Copenhagen, Denmark; 4 Copenhagen Affective Disorder Research Centre (CADIC), Psychiatric Centre Copenhagen, Copenhagen University Hospital, Copenhagen, Denmark; 5 Department of Psychology, University of Copenhagen, Copenhagen, Denmark

**Keywords:** activities of daily living, affective disorders, bipolar disorder, cognition, functioning, major depressive disorder, multimodal intervention

## Abstract

**Background:**

There is a compelling need for innovative intervention strategies for patients with affective disorders, given their increasing global prevalence and significant associated disability and impaired functioning. This study aimed to investigate whether a comprehensive multimodule individualized intervention (AWARE), targeting known mediators of functioning, improves functioning in affective disorders.

**Methods:**

AWARE was a randomized, controlled, rater-blind clinical trial conducted at two centers in the Capital Region of Denmark (Clinicaltrials.gov, NCT 04701827). Participants were adults with bipolar disorder or major depressive disorder and impaired functioning. Participants were randomized to the six-month AWARE intervention or treatment as usual (TAU). The AWARE intervention is based on the International Classification of Functioning, Disability and Health (ICF) Brief Core Set for Bipolar and Unipolar Disorder.

The primary outcome was observation-based functioning using the Assessment of Motor and Process Skills (AMPS). Secondary outcomes were functioning, QoL, stress, and cognition.

**Results:**

Between February 2021 and January 2023, 103 patients were enrolled; 50 allocated to AWARE treatment and 53 to TAU (96 included in the full analysis set). There was no statistically significant differential change over time between groups in the primary outcome (AMPS), however, both groups showed a statistically significant improvement at endpoint. The AWARE intervention had a statistically significant effect compared with TAU on secondary outcomes of patient-reported functioning, stress and cognition.

**Conclusion:**

Compared with TAU, the AWARE intervention was ineffective at improving overall functioning on the primary outcome, presumably due to the short duration of the intervention. Further development of effective treatments targeting functioning is needed.

## Introduction

The World Health Organization (WHO) ranks unipolar major depressive disorder (UD) and bipolar disorder (BD) among the leading causes of disability globally, with predictions of a further growing burden of these disorders (World Health Organization, 2017). The prevalence of UD is approximately 3–6%, and around 1% for BD (Kessing, 2020). The impact of affective disorders is considerable both from an individual and societal viewpoint, affecting many aspects of life and associated with both low quality of life, medical conditions, and a decreased lifespan of 8–12 years (Kessing, Ziersen, Andersen, & Vinberg, 2021). The impact on functional impairment is well established, not only during acute mood episodes and syndromic states but also during remission (Léda-Rêgo, Bezerra-Filho, & Miranda-Scippa, 2020). On the other hand, the effect of medical treatments on improving and restoring function is not as prominent as the effect on depressive symptoms (Kessing, Hansen, & Andersen, 2004; Sheehan, Nakagome, Asami, Pappadopulos, & Boucher, 2017).

In patients with affective disorders, there is cumulative evidence of functional impairment during remission (Burdick et al., 2022; Schwarz, Munkholm, Christensen, Kessing, & Vinberg, 2024b). This impairment can affect global functioning or specific domains such as occupational functioning, social functioning, or self-care (Chen, Fitzgerald, Madera, & Tohen, 2019). Dorland's illustrated medical dictionary defines global functioning as an ‘evaluation of a patient's functional level, including the ability to perform activities of daily living, done to prescribe or evaluate rehabilitation measures’ (Dorland, 2011). The WHO's International Classification of Functioning, Disability and Health (ICF) has been the international standard and framework to describe and measure functioning in patients with physical and psychiatric illness since 2001 (WHO, 2001). Functioning in the ICF is defined as ‘all body functions, activities and participation’, while ‘impairments, activity limitations and participation restrictions’ is defined broadly as ‘disability’ (WHO, 2001). Functioning can be assessed in various ways. Overall, functioning measures can be divided into three categories (Bonnín et al., 2019): (1) subjective (self-reported/patient-reported outcome) with self-administered questionnaires such as WHODAS 2.0 Questionnaire (Organization., 2010) and Sheehan Disability Scale (SDS) (Sheehan, 1986) (2) semi-objective (clinician-rated) interview based such as Global Assessment of Functioning (GAF) (American Psychiatric Association, 1994) and Functional Assessment Short Test (FAST) (Rosa et al., 2013) (3) objective (performance based) measures such as Assessment of Motor and Process Skills (AMPS) (Fisher & Jones, 2012) and University of California, San Diego Performance-Based Skills Assessment (UPSA) (Patterson, Goldman, McKibbin, Hughs, & Jeste, 2001). Subjective and semi-objective measures have mainly been utilized in studies of patients with affective disorders (Gitlin & Miklowitz, 2017; Sheehan et al., 2017).

It is critical for patients to obtain and consistently retain normal functioning during remission. Despite the general recognition of functioning as a central and meaningful outcome in affective disorders (Chen et al., 2019; McKnight & Kashdan, 2009), few studies have tested interventions for improving functioning as the primary outcome (Torrent et al., 2013), with most research mainly addressing cognition (Miskowiak et al., 2022). Although cognitive remediation trials show promising effects on cognitive functions, cognitive gains often fail to improve patients' real-live functioning due to limited transfer to real-world functioning, and investigation of the effects of multimodal interventions is recommended (Miskowiak et al., 2022; Samamé, Durante, Cattaneo, Aprahamian, & Strejilevich, 2023).

The primary aim of the present randomized controlled trial (RCT) study was therefore to investigate whether a multimodal comprehensive 360-degree intervention named Affective disorders: eliminate WArning signs And REstore functioning (AWARE) improve global functioning in patients with affective disorders. The hypothesis was that patients receiving the AWARE intervention would achieve greater improvement in functioning compared with patients receiving treatment as usual (TAU).

## Methods

### Study design

The study was a randomized open-label clinical trial, with two treatment arms and blinded outcome assessment (rater-blind). The study was conducted at two centers in the Capital Region of Denmark (Psychiatric Centre Copenhagen and Psychiatric Centre Northern Zealand). The authors assert that all procedures contributing to this work comply with the ethical standards of the relevant national and institutional committees on human experimentation and with the Helsinki Declaration of 1975, as revised in 2008. All procedures involving human subjects/patients were approved by The Regional Ethics Committee in the Capital Region of Denmark (protocol number H-20029748). All patients were provided oral and written information about the trial before written informed consent was obtained. Data permission was obtained from the Danish Data Protection Agency (j.nr. p-2020-1216). The study was registered at Clinicaltrials.gov. (NCT04701827) before inclusion started (registered on 8 January 2021) and a detailed study protocol has been published (Schwarz et al., 2022). A minor update in the trial registry was sent to Clinicaltrials.gov on 23 February 2022, regarding inclusion criteria age range (corrected from age 18–60 to 18–65) and target sample size (corrected from 140 to 120), as the original numbers were taken from an early protocol draft and did not reflect the final study design (as published in the final study protocol). The study is reported according to the Consolidated Standards of Reporting Trials (CONSORT) Statement (Moher et al., 2010). Patients were first recruited for the study on 1 February 2021.

### Participants

The patients were assessed and diagnosed by specialists in psychiatry according to the ICD-10 (World Health Organization, 2005) and were asked to participate if the clinicians found that their overall functioning was impaired.

Inclusion criteria were: Men or women, age 18–65 years with a diagnosis of BD or unipolar major depressive disorder by WHO International Classification of Disease 10th edition (ICD-10) diagnostic criteria; current state of remission or partial remission (defined as Hamilton Depression Rating Scale, 17-items (HDRS-17) (Hamilton, 1960) and Young Mania Rating Scale (YMRS) (Young, Biggs, Ziegler, & Meyer, 1978) scores of ⩽14); impaired functioning defined as a score ⩾11 according to the Functional Assessment Short Test (FAST) (Rosa et al., 2007); and ability to participate in two-thirds of the planned intervention visits. Exclusion criteria were: Severe physical disorder interfering with daily living; ongoing alcohol or substance abuse; electroconvulsive therapy treatment within the last 3 months before inclusion; dementia or inability to cooperate with the study, including inability to speak and read Danish. If patients did not fulfill the criteria for current state of remission or partial remission at initial screening, the patient was allowed further screenings during the inclusion period and was included if remission or partial remission was reached.

Sex/gender was collected as binary data (male and female). All participants gave written informed consent before participating in the study.

### Randomization and masking

The included patients were randomized with a 1:1 ratio to either (1) The intervention group with AWARE treatment + standard treatment/ TAU or (2) the control group with standard treatment/ TAU alone (TAU). Stratification was done for age (18–34 years *v.* 35–65 years), sex, and diagnosis (BD *v.* UD). Block randomization was used to ensure a balance in sample size across groups with randomly varied block sizes (2–6), and randomly varying order of treatment allocation within blocks. The random sequence was generated by an external clinical assistant, who did not participate in any other part of the study, and who was not involved in conducting the study. All participants were enrolled by the primary investigator (RS) and allocation was done in REDCap using the encoded random sequence (Harris et al., 2009). Due to the nature of the intervention the trial was an open-label design with no masking of allocation for the participants or the clinicians giving the intervention. The study was outcome assessor-blinded, and all outcome assessors were external assessors masked for allocation who did not participate in the treatment of participants or conducting the trial. Patients were carefully instructed not to disclose any information concerning their treatment allocation during endpoint assessments. FAST was the only outcome measure assessed unblinded (blinding of FAST was initially planned but due to practical reasons not possible).

### Procedures

Upon referral to the study, patients' diagnosis was confirmed with The Mini International Neuropsychiatric Interview (MINI) (Sheehan et al., 1998) and participants were assessed for eligibility according to the inclusion/exclusion criteria. Upon inclusion, the primary therapist (RS) implemented randomization in a consecutive manner, with participants being informed about their allocation. Baseline assessments were conducted in the week preceding the randomization. Blinded assessors carried out evaluations at endpoint (6 months after the randomization date). These assessments comprised an assessment of functioning, mood ratings, cognitive testing, and questionnaires addressing quality of life, subjective cognitive complaints, and functioning. A detailed description was published in the study protocol (Schwarz et al., 2022).

### The intervention

The manualized intervention has been described in detail elsewhere (Schwarz et al., 2022). In summary the AWARE intervention is a comprehensive multimodule individualized intervention, specifically targeting known mediators of functioning in these patients. The intervention is based on the International Classification of Functioning, Disability and Health (ICF) Brief Core Set for UD and BD (Ayuso-Mateos, Avila, Anaya, Cieza, & Vieta, 2013; Cieza et al., 2004). The five modules of the intervention targeted: ‘*1. ADL* (*Activities of Daily Living*) *ability as a part of carrying out daily routines; 2. Mood symptoms, medication and side effects; 3. Social, relatives and network; 4. Physical health, including Body Mass Index* (*BMI*), *biomarkers and exercise; 5. Cognition, circadian rhythm measured as sleep quality, and coping* (*stress reduction*)*’* (Schwarz et al., 2022).

Patients in the control group received TAU consisting of standard treatment in Denmark. Primary care outpatient treatment consists of treatments given by family doctors or private psychiatrists. Secondary care outpatient treatment can consist of treatment given by psychiatrist, psychologists, nurses, and social workers, as both group and individualized therapy and hospitalization. Treatments are public and free of charge.

### Outcomes

Primary outcome was change in Activities of Daily Living (ADL) motor and process ability from baseline to endpoint according to scores on the AMPS (Fisher & Jones, 2012). The AMPS is sensitive over time to changes in ADL ability and has a high inter-rater reliability, and a clinically relevant change in AMPS score is considered as 0.3 logic (Fisher & Jones, 2012). AMPS is a standardized observation-based assessment of two individually selected well known, meaningful and challenging ADL Tasks. The evaluation comprises scores for ADL Process skills (level of timeliness and organization skills) and ADL Motor skills (level of physical effort/clumsiness), with both scores also reflecting the safety and independence of the performance. A detailed description of the AMPS instrument is found in the study protocol (Schwarz et al., 2022). All AMPS assessments were conducted in the patient's homes.

Secondary outcomes were changes from baseline to endpoint in self-reported functioning using WHO Disability Assessment Schedule (WHODAS 2.0.) (World Health Organization, 2010) and FAST (Rosa et al., 2007), in quality of life and stress using WHO Quality of Life (WHOQOL) (Group, 1998) and Cohens Perceived Stress Scale (PSS) (Cohen, Kamarck, & Mermelstein, 1983), and cognition objectively measured with SCIP (Screen for Cognitive Impairment in Psychiatry) (Purdon, 2005) and Trail Making A and B (Reitan, 1992). Tertiary/other prespecified outcomes were patient reported cognition using Cognitive complaints in bipolar disorder rating assessment (COBRA) (Rosa et al., 2013) and functioning measured with the ADL-Interview (ADL-I) (Wæhrens, Kottorp, & Nielsen, 2021). An overview and description of assessment scales and instruments can be found in online Supplementary Table S3 in the Supplementary Materials.

Outcome assessments were done at six months by calibrated AMPS raters (primary outcome) and research-trained, non-specialist, medical doctors. Assessment of all outcomes and completion of participant self-reported questionnaires were made at baseline and endpoint (six months post-randomization).

### Safety assessment

Assessment of safety and adverse events was done systematically at months three and six of the intervention (mid-way and endpoint respectively) by query and registration of psychiatric hospitalizations.

### Statistical analysis

The statistical analyses were defined á priori and conducted using an intention-to-treat (ITT) approach (Schwarz et al., 2022). Differences in the primary and secondary endpoints were analyzed between the intervention and control group using an Analysis of Covariance (ANCOVA) model both unadjusted using only the baseline value of the endpoint as a covariate, and adjusted with sex, age, diagnosis and number of affective episodes as covariates. Outcome mean differences, 95% CIs, and *p* values are provided. Effect sizes were calculated as partial eta-squared (*η*^2^_p_). In addition, for the primary outcome, the change from baseline to endpoint (delta) was analyzed between groups, and the number of responders in each group was compared. Significance levels were set at *p* values of below 0.05. Statistical analysis was performed in IBM SPSS Statistics.

## Results

**Figure 1.**
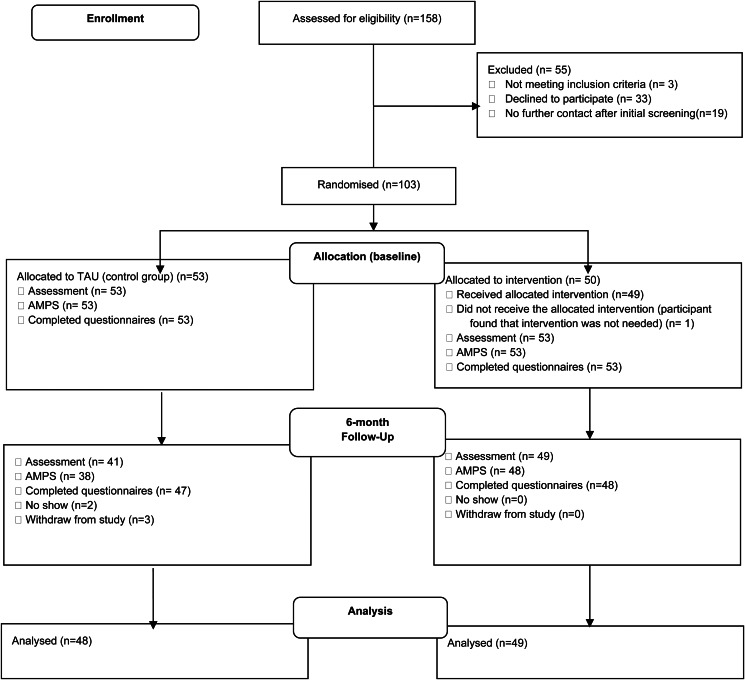
AWARE Trial CONSORT diagram. Legend: TAU, treatment as usual; AMPS, Assessment of Motor and Process Skills.

Between 1 February 2021, and 31 January 2023, 103 patients were enrolled and randomized. For full overview of screening, inclusion, and completion see Fig. 1. Sociodemographic and clinical characteristics are presented in Table 1. There were no statistically significant differences in baseline characteristics between the two groups.

**Table 1.** Sociodemographic and clinical characteristics of the included patients with bipolar or unipolar disorders according to treatment group

Randomization group	AWARE (*n* = 50)	TAU (*n* = 53)
Sociodemographic:		
Age, mean (s.d.)	41.64 (12.75)	39.92 (12.29)
Sex, female, *n* (%)	33 (66.00)	34 (64.15)
Education years, mean (s.d.)	13.61 (2.40)	13.83 (2.75)
Marital status yes, *n* (%)	15 (30.00)	14 (26.42)
Employment status ‘employed’, *n* (%)	11 (22.00)	15 (28.30)
Disability Pension, *n* (%)	10 (20.00)	3 (5.66)
Student, *n* (%)	4 (8.00)	9 (16.98)
Diagnosis:		
Unipolar, *n* (%)	15 (30.00)	17 (32.08)
Bipolar, *n* (%)	35 (70.00)	36 (67.92)
Age at illness onset, mean (s.d.)	20.38 (8.30)	20.34 (8.57)
Number of episodes:		
Depressive, median [IQR]	10 [13]	9 [11]
Manic (only for Bipolar 1), median [IQR]	2 [4]	1 [4]
Hypomanic (only for Bipolar 1 and 2), median [IQR]	10 [21]	10 [10]
Clinical:		
Time in remission/partial remission months, mean (s.d.)	6.48 (9.64)	5.96 (8.06)
HDRS-17 total score, mean (s.d.)	9.48(3.41)	9.75 (2.85)
YMRS total score, mean (s.d.)	2.40 (2.29)	2.98 (2.51)
Psychiatric comorbidity, *n* (%)	10 (20.00)	13 (24.53)
Previous suicide attempts, *n* (%)	8 (16.00)	16 (30.19)
Previous received ECT, *n* (%)	7 (14.00)	3 (5.66)
Previous hospitalization, *n* (%)	31 (62.00)	24 (45.28)
Number of hospitalizations, median [IQR]	1 [3]	0 [2]
FAST total, mean (s.d.)	37.76 (10.06)	35.53(10.22)
SCIP total, mean (s.d.)	69.56 (15.18)	67.83 (12.35)
Body mass index, mean (s.d.)	29.09 (6.84)	29.01 (6.89)
Medical comorbidity (yes), *n* (%)	28 (56.00)	22 (41.51)
Current medication:		
Lithium, *n* (%)	19 (38.00)	21 (39.62)
Antipsychotics, *n* (%)	26 (52.00)	22 (41.51)
Antidepressants, *n* (%)	21 (42.00)	22 (41.51)
Anticonvulsics, *n* (%)	27 (54.00)	26 (49.06)
Benzodiazepines, *n* (%)	16 (32.00)	11 (20.75)
Others, *n* (%)	34 (68.00)	34 (64.15)

AWARE, Affective disorders: eliminate WArning signs And REstore functioning; TAU, treatment as usual; IQR, Interquartile Range; HDRS-17, The 17-item Hamilton Depression Rating Scale; YMRS, Young Mania Rating Scale; ECT, Electroconvulsive Therapy; FAST, Functioning Assessment Short Test; SCIP, Screen for Cognitive Impairment in Psychiatry.

The overall dropout rate of both treatment arms was low, with no statistically significant difference between the two groups (Fishers Exact Test, *p* = 0.206); AWARE 2.0% (1/50) and TAU 9.4% (5/53), however, the number of patients assessed for the primary outcome (AMPS) at endpoint in the AWARE arm was significantly higher; 96.0% (48/50), compared with the TAU arm; 71.7% (38/53) (Fishers Exact Test, *p* = 0.001).

Online Supplementary Table S4, supplementary materials, shows the number of AWARE intervention sessions given to the intervention group, as well as the content of other psychiatric treatments the patients in the intervention and control group received during the trial. There was no difference in the content of other psychiatric treatments received by the intervention and control groups during the trial.

### Primary outcome

The ANCOVA analysis addressing the treatment effect of the AWARE intervention compared with TAU on the primary outcome showed no statistically significant differential change over time between the two groups. The unadjusted model including only baseline AMPS scores as a covariate, to account for the difference in treatment effect from baseline to endpoint, showed no significant mean difference between groups on AMPS process of 0.07 (CI 95 −0.11 to −0.26, *p* = 0.434) and no significant mean difference on AMPS motor of 0.06 (CI 95 −0.17 to −0.291, *p* = 0.590). Adjusting for age, sex, diagnosis and number of affective episodes still revealed no statistically significant differences, with an adjusted mean difference on AMPS process of 0.10 (CI 95 −0.10 to −0.29, *p* = 0.319) and an adjusted mean difference on AMPS motor of 0.11 (CI 95 −0.11 to −0.33, *p* = 0.3).

### Exploratory analysis on the primary outcome

**Figure 2.**
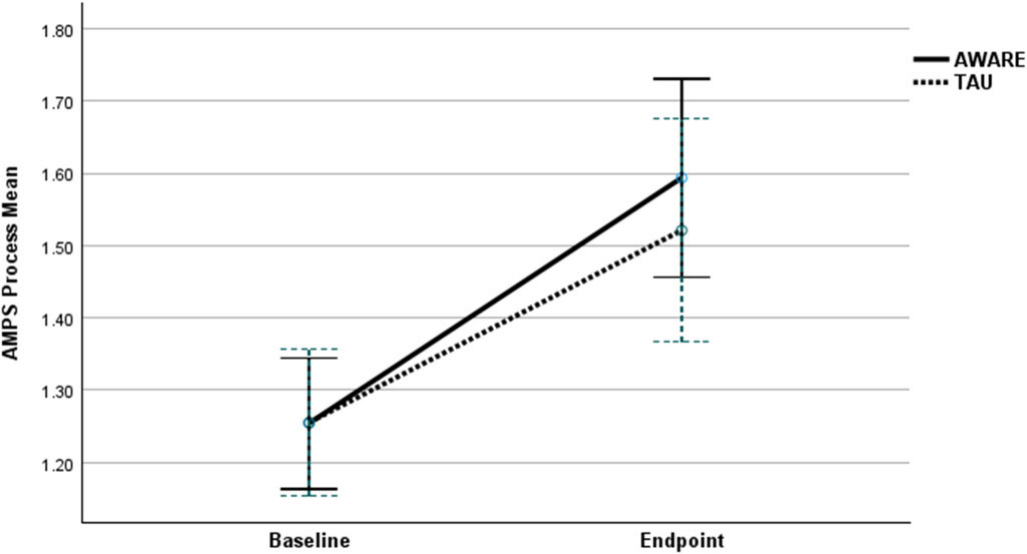
Changes in mean AMPS (Assessment of Motor and Process Skills) process scores (with 95% confidence intervals) from baseline to endpoint according to treatment group (the active Affective disorders: eliminate WArning signs And REstore functioning (AWARE) and the treatment as usual (TAU) Group.

The number of responders in each group (defined as a clinically relevant improvement of ⩾0.3 logit from baseline to endpoint on AMPS) was 26 (54.2%) in the AWARE group and 18 (47.4%) in the TAU group, with no statistically significant difference between groups (Pearson χ^2^ *p* = 0.531). When analyzing the change in AMPS score from baseline to endpoint individually in each group using a paired *t* test, both groups showed a statistically significant and clinically relevant improvement (AWARE: Mean difference 0.34 CI 95 0.46–0.21, *p* < 0.001 and TAU: Mean difference 0.34 CI 95 0.51–0.18, *p* < 0.001). The change in unadjusted AMPS Process scores from baseline to endpoint is graphically presented in Fig. 2. We conducted four exploratory sub-analyses in relation to the primary outcome, stratifying on age group, disability pension status, cognitive impairment, and baseline HDRS score.

Stratifying on cognitive impairment at baseline (SCIP total score below 70, AWARE *n* = 21, TAU *n* = 17) showed a significant and clinically relevant effect of the AWARE intervention *v.* TAU on AMPS Process in both unadjusted (*B* = 0.33, CI 95 0.02–0.64 *p* = 0.038) and adjusted models (*B* = 0.42, CI 95 0.10–0.74 *p* = 0.013) in the cognitively impaired group. The effect sizes were *η*^2^_p_ = 0.12 (medium effect size >0.06) and *η*^2^_p_ = 0.18 (large effect size >0.14) respectively. There were no significant results on any of the models stratifying on age group, disability pension status and baseline HDRS score (results not presented in detail).

### Secondary outcomes

The secondary outcome analyses between the AWARE and TAU group are presented in Table 2. There was a statistically significant difference on the perceived stress scale (PSS) −2.86 (CI 95 −5.54 to −0.19, *p* = 0.036) between the two groups in the model adjusting for baseline value, and still present in the model also adjusting for age, sex, diagnosis, and number of depressive episodes; −2.89 (CI 95 −5.58 to −0.207, *p* = 0.04). The effect size was *η*^2^_p_ = 0.047 and *η*^2^_p_ = 0.049, respectively (small effect sizes <0.06). Further, there was a statistically significant differential difference in processing speed (measured by Trail Making A) of −7.24 (CI 95 −13.72 to −0.77, *p* = 0.029) between the two groups in the model adjusting for age, sex, diagnosis and number of depressive episodes (small to medium effect size, *η*^2^_p_ = 0.06). Finally, there was a statistically significant differential change in functioning measured by FAST (see, Table 2) with a large effect size (effect size Model 1; *η*^2^_p_ = 0.149, Model 2; *η*^2^_p_ = 0.0158). There were no statistically significant differential changes between groups on remaining secondary outcomes, neither on sub-domains of WHODAS and SCIP (results not presented in detail).

**Table 2.** Results of the secondary and tertiary outcome measures at six-months follow-up (endpoint) in patients with bipolar or unipolar disorders according to treatment group

	Model 1^a^			Model 2^b^		
Secondary outcome	Difference between AWARE and TAU	95% CI	*p*	Difference between AWARE and TAU	95% CI	*p*
WHODAS	−3.36	−8.75 to 2.03	0.219	−3.96	−9.19 to 1.27	0.136
WHOQoL Physical^c^	1.56	−4.36 to 7.47	0.603	1.93	−3.90 to 7.76	0.512
WHOQoL Psych^c^	0.41	−5.95 to 6.76	0.899	1.07	−5.24 to 7.38	0.736
WHOQoL Social^c^	1.27	−5.24 to 7.79	0.699	1.49	−5.07 to 8.04	0.653
WHOQoL Environment^c^	1.86	−3.12 to 6.83	0.46	2.32	−2.68 to 7.32	0.359
PSS	−2.86	−5.54 to −0.17	0.036	−2.89	−5.58 to −0.21	0.035
FAST^d^	−7.13	−10.76 to −3.49	0.001	−7.27	−10.94 to −3.61	0.001
SCIP	0.45	−2.93 to 3.82	0.794	0.87	−2.50 to 4.24	0.609
Trail Making A	−6.27	−12.79 to 0.26	0.06	−7.24	−13.72 to −0.77	0.029
Trail Making B	10.35	−3.67 to 24.36	0.146	8.5	−5.77 to 22.77	0.239
Tertiary outcome						
ADLI^c^	0.08	−0.36 to 0.51	0.727	0.17	−0.27 to 0.62	0.445
COBRA	−2.55	−5.10 to 0.00	0.05	−2.8	−5.37 to −0.23	0.033

AWARE, Affective disorders: eliminate WArning signs And REstore functioning; TAU, treatment as usual; WHODAS, World Health Organization Disability Assessment Schedule 2.0; WHOQoL, World Health Organization Quality of Life; PSS, Cohen's Perceived Stress Scale; Functional Assessment Short Test (FAST); SCIP, Screen for Cognitive Impairment in Psychiatry; ADLI, Activities Of Daily Living Interview; COBRA, Cognitive complaints in bipolar disorder rating assessment.

a Model 1: Analysis of Covariance, unadjusted (only adjusted for baseline value).

b Model 2: Analysis of Covariance, adjusted for age, sex, diagnosis and number of affective episodes (and adjusted for baseline value).

c Indicates that a large score is favorable to the patient.

d Unblinded outcome assessment.

### Tertiary outcomes

Other prespecified outcome measures were ADL-I (no significant difference between groups) and COBRA (significant difference in adjusted model; *p* = 0.033, with a small effect size; *η*^2^_p_ = 0.051). Unadjusted means for sociodemographics, clinical status and outcome measures at follow-up/endpoint are presented in online Supplementary Table S6 in Supplementary Materials.

### Adverse events/safety

Spontaneously reported adverse events were recorded at a visit midway through the treatment program (3 months) and at endpoint. No participants reported adverse events associated with the treatment, and the AWARE treatment group had a high completion of 98.0% (49/50), which further indicates treatment safety. The AWARE and TAU groups did not differ in the number of patients hospitalized during the intervention period (AWARE; 4/49 [8.2%], TAU 4/42 (9.6%), Pearson χ^2^ *p* = 0.819). Affective episode relapse rates were similar during the intervention period in the AWARE group (18/41 [47%]) and TAU group (21/40 [53%]).

### Drop out analysis

The trial had an overall low dropout rate, however fewer patients in the control group participated in the assessment of the primary outcome at endpoint. A dropout analysis comparing baseline characteristics of the patients participating in the assessment of the primary outcome at endpoint *v.* patients who did not was made for the control group. The results are presented in online Supplementary Table S5 in the Supplementary Materials. The number of patients with a history of previous psychiatric hospitalization was significantly lower in the patients not participating in the assessment of AMPS at endpoint (10 patients [66.6%] *v.* 14 [36.8%], Pearson χ^2^ *p* = 0.049). The non-completers had a lower SCIP score at baseline (61.40 s.d. 11.70) compared with the completers (70.37 s.d. 11.80). All remaining differences in baseline characteristics did not reveal statistically significance. The dropouts were also compared on selected endpoint outcomes/measures, however data on all these outcomes was not available for all 15 participants. The scores on HDRS were significantly higher in the dropouts (14.80 s.d. 5.76) *v.* completers (9.19 s.d. 5.57) (*p* = 0.042). There was no statistically significant difference on remaining endpoints. As data on AMPS at endpoint only was missing for two patients (4.0%) in the intervention group, a statistical comparison of these two participants was not carried out.

### Post hoc statistical power analysis

The trial included 103 patients, which was less than the calculated sample size of 120 patients in the protocol (Schwarz et al., 2022). Despite intense recruiting efforts (the study was conducted during three lock downs due to COIVD 19) and an extension of the recruitment period, the desired sample size was not achieved. However, post hoc statistical power analysis confirmed sufficient power to detect the minimum clinically important difference in the primary outcome (o.3 logit on AMPS). The power was sufficient (>80%) to detect a significant group difference (*p* < 0.05).

## Discussion

This randomized open label outcome assessor blinded clinical trial study investigated the effects of a multimodule individualized six months intervention on functioning in 103 patients with unipolar depressive disorder and bipolar disorder in full or partial remission at baseline, with impaired functioning. Patients were randomized to either the AWARE intervention or TAU. Compared with TAU, we did not confirm the hypothesized effects on our primary outcome measure, AMPS, in improving daily functioning in the intervention group. Both the intervention group and TAU group did, however, improve significantly in observer-based functioning (AMPS score) from baseline to endpoint. Patients in the active group showed a significant improvement in the non-blinded secondary outcome measure of self-reported functioning (FAST score) relative to TAU, which was of a large effect size. They also reported a significant reduction in perceived stress and improved self-reported cognitive functions compared with TAU with small effect sizes. Finally, patients in the active group showed improvement in an objective measure of attention (TMT-A). The intervention did not affect quality of life or improved the overall cognitive scores (SCIP).

Notably, post hoc sensitivity analysis revealed a statistically and clinically significant effect of the AWARE intervention in the patients with cognitive impairment (measured on SCIP) at baseline. Strategy-based cognitive remediation was a central part of the intervention program, and it is plausible that patients with cognitive impairment would benefit from the intervention.

### Comparison with findings from other studies

The largest study with functioning as the primary outcome (conducted in Spain) found an effect of a two-year functional remediation program compared with TAU in patients with BD (primary outcome FAST) (Torrent et al., 2013). This study mainly differed from our present trial by having a less severely ill population due to rigorous inclusion criteria (clinical remission for three months before entering the trial and HDRS-17 score of ⩽8). It is worth noting that the trial compared functional remediation with both TAU and psychoeducation, with the effect of functional remediation not being superior to psychoeducation. As a psychoeducational program is a central part of the standard treatment given in Denmark, the control group (TAU) in our trial may have benefitted from this, contributing to the presents study lack of effect on the primary outcome AMPS. The effect on FAST found in the Spanish study (Torrent et al., 2013), was however replicated in the present study. Another study testing a more integrative approach similar to the AWARE intervention was performed in 2020, but the trial was stopped prematurely due to the SARS-CoV-2 crisis(Valls et al., 2021). Future studies replicating the effect of functional remediation are currently planned (Accardo et al., 2023; Zyto, Jabben, Schulte, Regeer, & Kupka, 2016).Trials of cognitive remediation programs have shown varying degrees of effectiveness on functional outcomes (Gomes et al., 2019; Vicent-Gil et al., 2022). The studies reporting positive effects on functional outcomes have used interview-based or patient-reported measures of functioning (Torrent et al., 2013; Valls et al., 2021; Vicent-Gil et al., 2022). These instruments rely to a large degree on information reported or relayed by the patient, which could raise a question about the accuracy of the individual, both regarding insight and mood symptoms (Sheehan et al., 2017). Using an observer-based measure of functioning as the primary outcome in our study, might limit the potential for significant improvement compared with patient-reported measures, as patients have been found to be more severely functionally impaired on observer-based measures (Decker, Waehrens, Miskowiak, & Vinberg, 2017).

### Strengths and limitations

The study has several limitations: (1) The limited duration of the intervention, with six months as possibly too short a span to implement treatment strategies on different areas of functioning and improve overall functioning (2) The participants had severe functional and cognitive impairment at baseline, as seen from FAST and SCIP baseline scores, thus requiring extensive intervention regarding many areas of functioning. Furthermore, the severity and length of the disorders are considerable, including participants with many previous mood episodes (ref. Table 1), which is associated with poor response to interventions (Scott et al., 2006) (3) The pragmatic nature of the study yields high generalizability regarding patients with affective disorders and functional impairment, however the broad inclusion of severely functional impaired participants with both psychiatric and medical comorbidities, may limit the potential for considerable improvement in the patients. The higher number of patients on disability pension in the AWARE group (20.00% *v.* 5.66% in TAU), may also have limited the potential for improvement among these patients, as their motivation might be lower. Regarding the primary outcome, we do not think this as a false negative finding caused by low power (type II error), as the obtained sample size was larger than needed based on post hoc power calculations. Power calculations were however not based on secondary outcomes. The blinded outcome assessment is a prominent strength, as it reduces bias. The lack of blinding in the assessment of FAST somehow limits a more directs comparison between our study and previous trials on this measurement (Léda-Rêgo et al., 2020; Torrent et al., 2013), as unblinded assessment is associated with a high risk of potential bias.

A considerable strength of the study is the comprehensive assessment and intervention, targeting the most well-known factors contributing to impaired functioning. Furthermore, functioning was assessed as both observer/performance-based measures of ADL ability (AMPS) as part of functioning conducted in the patientś homes and thereby everyday life as well as patient-reported (WHODAS) and clinician-rated/interview-based (ADL-I and FAST). Most studies only measure functioning with one of these modalities, which might limit the validity of the results, as each modality has its weaknesses and negative attributes (Sheehan et al., 2017). However, the use of AMPS assessment/observations in patients' home surroundings at baseline and endpoint was the primary cause of why referred patients declined to participate in the trial. Measuring functioning with AMPS also caused dropout/missing data on this outcome, as several participants who took part in the endpoint assessment were unwilling to complete the AMPS observation at the endpoint. The patients who did not participate in AMPS at the endpoint had lower SCIP scores at baseline and higher HDRS scores at the endpoint, pointing to these patients having a higher disease burden at the time of the study. The various existing methods for assessing functioning in clinical trials each have inherent advantages and disadvantages. Questionnaires can be easily applied but reporting relies strictly on the patient́s insight and credibility. Clinician-rated measures based on interviews are less subjected to patients' negative cognitive bias but require time and training of clinicians, and the impact of the quality of reporting by the patient cannot be denied. As patients in the present study and comparable studies are not blinded to treatment allocation, reporting bias may be present, with patients who received the intervention tending to report outcomes more optimistically at follow-up (Hróbjartsson, Kaptchuk, & Miller, 2011). The effect reported in studies using patients-reported and interview-based functional outcomes might overestimate the positive impact in the intervention group due to reporting bias (even clinician-rated scales, such as FAST, rely largely on the patients' perception). Performance-based measures such as AMPS, based on standardized observations conducted by a trained clinician (when using AMPS, an occupational therapist) can be applied in a highly standardized manner. However, this assessment method is time-consuming, logistically challenging, and requires training of the assessors. Performance-based outcomes thus have the advantage of eliminating the risk of reporting bias. Compared with other outcome measures, such as cognitive outcomes, functional measures benefit from being more generalizable to real-world functioning and seek to directly address the issues of daily life the patient is experiencing.

### Implications

Despite the negative result of the primary outcome, the trial has implications regarding future research. First and foremost, the study highlights an unmet need for effective interventions targeting functioning in patients with affective disorders, even in patients with low levels of mood symptoms (defined as full or partial remission on HDRS-17). Only a few previous RCT intervention studies have used functioning as the primary outcome (Torrent et al., 2013; Valls et al., 2021). The multimodule individualized AWARE intervention was not superior to TAU on improving observer-based functioning. Nevertheless, patient-reported measures showed positive effects, indicating that participants found the intervention relevant and helpful.

Further, the sensitivity analysis showed an effect of the AWARE intervention compared with TAU in patients with cognitive impairment at baseline, supporting a recent meta-analysis calling for multimodal interventions aiding transfer to real-world functioning in patients with objective cognitive performance deficits (Miskowiak et al., 2022). The positive results seen in the patients with cognitive impairment suggest that patients with cognitive impairment as the primary cause of functional impairment might be more responsive to treatment, whereas functional impairment caused by more complex mechanisms, such as inherent behavioral patterns and a history of childhood trauma, might be even more difficult to treat (Schwarz, Miskowiak, Kessing, & Vinberg, 2024a). Functioning is an essential and relevant outcome in patients with affective disorder, and further development of effective treatments and treatment strategies explicitly targeting functioning are needed. The present trial finds possible beneficial effects of a multimodal approach in patients with functional impairment coinciding with cognitive impairment. Based on the current finding, future trials/interventions targeting functioning should have a duration of more than six months.

## Supporting information

Schwarz et al. supplementary material Schwarz et al. supplementary material

## Data Availability

The data and analytic code supporting the findings of this study are available from the corresponding author (RS) upon reasonable request.

## Supplementary material

The supplementary material for this article can be found at https://doi.org/10.1017/S0033291724002526
