# Supplementary material for: Affective disorders: eliminate WArning signs And REstore functioning: AWARE. Results from a randomized controlled multimodular intervention study targeting functioning in patients with affective disorders
Source: Psychol Med. 2024 Oct 31;54(14):3985–94. doi: 10.1017/S0033291724002526 (PMC11578905; doi:10.1017/S0033291724002526)
Supplement: Schwarz et al. supplementary material [file S0033291724002526sup001.docx]

Supplementary Materials

Table of Contents

1. ***Table 3 Overview of variables and measurements …………………………………………………2***
2. ***Table 4 Content of treatment during the intervention period for completers of the study in the AWARE and TAU group……………………………………………………………………………3***
3. ***Table 5 Comparison of baseline sociodemographic and clinical variables between patients in TAU with and without study dropout on primary endpoint (AMPS)……………………………………4***
4. ***Table 6 Data on sociodemographics, clinical status and outcome measures at***

***follow-up/endpoint………………………………………………………………………………….6***

| **Table 3 Overview of variables and measurements** | | | |
| --- | --- | --- | --- |
| Variables | Measurements | Assessment method | Total score range and interpretation |
| Activities of daily living (observer/performance- based functioning) | AMPS (Process) | Observation-based (performance based) | (-4)-3  Higher scores = better functioning |
|  | AMPS (Motor) | Observation-based (performance based) | (-3)-4  Higher scores = better functioning |
| Patient-reported functioning | WHODAS | Questionnaire | 0-100  Higher scores = worse functioning |
| Clinician rated/interview based functioning | FAST | Interview | 0-72  Higher scores = worse functioning |
|  | ADL-I | Interview | (-5.84)-6.41  Higher scores = better functioning |
| Patient reported stress | PSS | Questionnaire | 0-40  Higher scores = more stress |
| Quality of life | WHOQOL | Questionnaire | 0-100^a^  Higher scores = better quality of life |
| Cognitive performance | SCIP | Performance based | 0-Ꚙ^b^  Higher scores = better cognitive performance |
|  | Trail Making Test A and B | Performance based | 0^c^-600  Higher scores = better cognitive performance |
| Patient-reported cognition | COBRA | Questionnaire | 0-48  Higher scores = more cognitive impairment |
| Depressive symptoms severity | HDRS-17 | Interview | 0-52  Higher scores = more symptoms |
| Manic symptoms severity | YMRS | Interview | 0-60  Higher scores = more symptoms |
| AMPS, Assessment of Motor and Process Skills; WHODAS, World Health Organization Disability Assessment Schedule 2.0; FAST, Functional Assessment Short Test; ADLI, Activities Of Daily Living Interview; PSS, Cohen’s Perceived Stress Scale; WHOQoL, World Health Organization Quality of Life; SCIP, Screen for Cognitive Impairment in Psychiatry; COBRA, Cognitive complaints in bipolar disorder rating assessment; HDRS-17, The 17-item Hamilton Depression Rating Scale; YMRS, Young Mania Rating Scale. | | | |
| a. Score is calculated for each of the four domains, with no total score combining the domains | | | |
| b. No theoretical upper range | | | |
| c. Theoretical lower range |  |  |  |

| **Table 4 Content of treatment during the intervention period for completers of the study in the AWARE and TAU group** | | |  |
| --- | --- | --- | --- |
|  | AWARE (n=49) | TAU (n=46) | p= (two-sided) |
| AWARE sessions completed, median [IQR] (Range) | 12 [4] (6-18) | - |  |
| Patients treated in psychiatric secondary care setting, n (%) | 40 (81.63) | 35 (76.09) | 0.508 |
| Number of secondary care visits/sessions, median [IQR] (Range) | 6 [10] (0-24) | 10 [12] (0-31) | 0.477 |
| Number of secondary care phone consultations, median [IQR] (Range) | 1 [2] (0-15) | 1 [2] (0-12) | 0.846 |
| Patients treated in psychiatric primary care setting, n (%) | 6 (12.24) | 7 (15.22) | 0.674 |
| Patients not treated in psychiatric primary or secondary care settings, n (%) | 2 (4.08) | 0 | 0.166 |
| AWARE, Affective disorders: eliminate WArning signs And REstore functioning; TAU, Treatment As Usual; IQR, Interquartile Range | | | |

| **Table 5 Comparison of baseline sociodemographic and clinical variables between patients in TAU with and without study dropout on primary endpoint (AMPS)** | | | |
| --- | --- | --- | --- |
|  | Dropuot n=15 | Completion n=38 | p= (Two-sided) |
| Age, mean (s.d.) | 42.20 (14.05) | 39.03 (11.61) | 0.201^a^ |
| Sex, female, n (%) | 8 (53.33) | 26 (68.42) | 0.302^b^ |
| Education years, mean (s.d.) | 13.20 (2.66) | 14.08 (2.77) | 0.298^a^ |
| Marital status yes, n (%) | 3 (20,00) | 11 (28.95) | 0.732^c^ |
| Employment status "employed", n (%) | 4 (26.67) | 11 (28.95) | 0.868^c^ |
| **Diagnosis:** |  |  |  |
| Unipolar, n (%) | 5 (33.33) | 12 (31.58) | 0.902^b^ |
| Bipolar, n (%) | 10 (66.67) | 26 (68.42) | 0.902^b^ |
| Age at illness onset, mean (s.d.) | 19.47 (9.60) | 20.68 (8.24) | 0.646^a^ |
| **Number of episodes:** |  |  |  |
| Depressive, median [IQR] | 9 [14] | 9 [11] | 0.931^a^ |
| Manic (only for Bipolar 1), median [IQR] | 2 [4] | 1 [8] | 0.567^a^ |
| Hypomanic (only for Bipolar 1 and 2), median [IQR] | 10 [14] | 10 [10] | 0.767^a^ |
| **Clinical:** |  |  |  |
| Time in remission/partial remission months, mean (s.d.) | 4.80 (5.20) | 6.42 (8.96) | 0.515^a^ |
| HDRS-17 total score, mean (s.d.) | 10.00 (2.83) | 9.66 (2.90) | 0.698^a^ |
| YMRS total score, mean (s.d.) | 2.87 (2.45) | 3.03 (2.56) | 0.837^a^ |
| Psychiatric comorbidity, n (%) | 3 (20.00) | 10 (26.32) | 0.736^c^ |
| Previous suicide attempts, n (%) | 5 (33.33) | 11 (28.95) | 0.754^b^ |
| Previeous received ECT, n (%) | 1 (6.67) | 2 (5.26) | 0.842^c^ |
| Previeous hospitalisation, n (%) | 10 (66.67) | 14 (36.84) | 0.049^b^ |
| Number of hospitalisations, median [IQR] | 1 [3] | 0 [1] | 0.097^a^ |
| FAST total, mean (s.d.) | 38.07 (9.33) | 34.52 (10.50) | 0.260^a^ |
| SCIP total, mean (s.d.) | 61.40 (11.70) | 70.37 (11.80) | 0.016^a^ |
| Body mass index, mean (s.d.) | 29.38 (5.19) | 28.87 (7.52) | 0.811^a^ |
| Medical comorbidity (yes), n (%) | 7 (46.67) | 24 (63.16) | 0.272^b^ |
| WHODAS, mean (s.d.) | 37.55 (17.56) | 40.22 (15.74) | 0.592^a^ |
| PSS, mean (s.d.) | 21.53 (4.61) | 20.87 (6.40) | 0.716^a^ |
| **Current medication:** |  |  |  |
| Lithium, n (%) | 6 (40.00) | 14 (36.84) | 0.831^b^ |
| Antipsychotics, n (%) | 6 (40.00) | 16 (42.10) | 0.889^b^ |
| Antidepressants, n (%) | 6 (40.00) | 16 (42.10) | 0.889^b^ |
| Anticonvulsics, n (%) | 7 (46.67) | 19 (50.00) | 0.827^b^ |
| Benzodiazepines, n (%) | 1 (6.67) | 10 (26.32) | 0.149^c^ |
| Others, n (%) |  |  |  |
| **AMPS baseline** |  |  |  |
| Process, mean (s.d.) | 1.24 (0.34) | 1.26 (0.27) | 0.862^a^ |
| Motor, mean (s.d.) | 2.17 (0.32) | 2.09 (0.33) | 0.441^a^ |
| **Endpoint** |  |  |  |
| FAST total, mean (s.d.)^d^ | 45.40 (8.73)^e^ | 34.75 (14.02)^f^ | 0.108^a^ |
| HDRS-17 total score, mean (s.d.)^d^ | 14.80 (5.76)^e^ | 9.19 (5.57)^f^ | 0.042^a^ |
| WHODAS, mean (s.d.)^d^ | 37.92 (17.92)^g^ | 34.70 (16.35)^h^ | 0.590^a^ |
| PSS, mean (s.d.)^d^ | 21.70 (6.80)^g^ | 20.11 (6.90)^h^ | 0.519^a^ |
| TAU, Treatment As Usual; IQR, Interquartile Range; HDRS-17, The 17-item Hamilton Depression Rating Scale; YMRS, Young Mania Rating Scale; ECT, Electroconvulsive Therapy; FAST, Functioning Assessment Short Test; SCIP, Screen for Cognitive Impairment in Psychiatry; WHODAS, World Health Organization Disability Assessment Schedule 2.0; PSS, Cohen’s Perceived Stress Scale; AMPS, Assessment of Motor and Process Skills. | | | |
| a. independent samples t-test |  |  |  |
| b. Pearson chi-square |  |  |  |
| c. Fishers Exact Test |  |  |  |
| d. score at endpoint |  |  |  |
| e. based on n=5 |  |  |  |
| f. based on n=36 |  |  |  |
| g. based on n=10 |  |  |  |
| h. based on n=37 |  |  |  |

| **Table 6 Data on sociodemographics, clinical status and outcome measures at follow-up/endpoint** | | |
| --- | --- | --- |
|  | AWARE n=49 | TAU n=47 |
| **Sociodemographic:** |  |  |
| Employment status "employed", n (%) | 12 (24.49) | 10 (24.39)^a^ |
| **Number of episodes:** |  |  |
| Depressive, median [IQR] | 11 [13] | 11 [13]^b^ |
| Manic (only for Bipolar 1), median [IQR] | 2 [4]^c^ | 1 [5]^d^ |
| Hypomanic (only for Bipolar 1 and 2), median [IQR] | 13 [20]^e^ | 10 [11]^f^ |
| **Clinical:** |  |  |
| Time in remission/partial remission months, mean (s.d.) | 6.44 (7.25) | 5.83 (6.44)^b^ |
| HDRS-17 total score, mean (s.d.) | 9.55 (6.43) | 9.88 (5.82)^a^ |
| YMRS total score, mean (s.d.) | 2.02 (2.45) | 2.15 (2.99)^a^ |
| Previeous hospitalisation, n (%) | 30 (61.22) | 18 (42.86)^b^ |
| Number of hospitalisations, median [IQR] | 1 [3] | 0 [2]^b^ |
| FAST total, mean (s.d.) | 31.04 (12.97) | 36.05 (13.86)^a^ |
| SCIP total, mean (s.d.) | 71.11 (15.57)^g^ | 68.97 (14.65)^h^ |
| Body mass index, mean (s.d.) | 28.73 (7.12)^i^ | 28.90 (6.86)^j^ |
| **Current medication:** |  |  |
| Lithium, n (%) | 17 (34.69) | 18 (39.13)^k^ |
| Antipsychotics, n (%) | 24 (48.98) | 15 (32.61)^k^ |
| Antidepressants, n (%) | 21 (42.86) | 18 (39.13)^k^ |
| Anticonvulsics, n (%) | 28 (57.14) | 17 (36.96)^k^ |
| Benzodiazepines, n (%) | 15 (30.61) | 10 (21.74)^k^ |
| Others, n (%) | 33 (67.35) | 28 (60.87)^k^ |
| **Outcomes** |  |  |
| AMPS Process, mean (s.d.) | 1.59 (0.46)^i^ | 1.52 (0.49)^h^ |
| AMPS Motor, mean (s.d.) | 2.32 (0.61)^i^ | 2.33 (0.49)^h^ |
| WHODAS, mean (s.d.) | 36.22 (19.92)^i^ | 35.39 (16.55) |
| WHOQoL Physical, mean (s.d.) | 57.22 (19.03)^i^ | 59.35 (16.31) |
| WHOQoL Psych, mean (s.d.) | 43.23 (21.64)^i^ | 43.88 (17.29) |
| WHOQoL Social, mean (s.d.) | 53.30 (22.00)^i^ | 49.29 (22.44) |
| WHOQoL Environment, mean (s.d.) | 67.06 (18.45)^i^ | 64.23 (16.14) |
| PSS, mean (s.d.) | 19.13 (8.27)^i^ | 20.45 (6.84) |
| ADLI, mean (s.d.) | 2.68 (1.05)^i^ | 2.70 (1.21)^h^ |
| Trail Making A, mean (s.d.) | 36.00 (26.83)^g^ | 37.66 (19.91)^h^ |
| Trail Making B, mean (s.d.) | 95.11 (82.15)^g^ | 84.57 (38.81)^h^ |
| AWARE, Affective disorders: eliminate WArning signs And REstore functioning; TAU, Treatment As Usual; IQR, Interquartile Range; HDRS-17, The 17-item Hamilton Depression Rating Scale; YMRS, Young Mania Rating Scale; FAST, Functioning Assessment Short Test; SCIP, Screen for Cognitive Impairment in Psychiatry; AMPS, Assessment of Motor and Process Skills; WHODAS, World Health Organization Disability Assessment Schedule 2.0; WHOQoL, World Health Organization Quality of Life; PSS, Cohen’s Perceived Stress Scale; ADLI, Activities Of Daily Living Interview. | | |
| a. based on n=41 |  |  |
| b. based on n=42 |  |  |
| c. based on n=10 |  |  |
| d. based on n=9 |  |  |
| e. based on n=34 |  |  |
| f. based on n=30 |  |  |
| g. based on n=47 |  |  |
| h. based on n=38 |  |  |
| i. based on n=48 |  |  |
| j. based on n=39 |  |  |
| k. based on n=46 |  |  |
